# Decreased Secondary Lesion Growth and Attenuated Immune Response after Traumatic Brain Injury in *Tlr2/4^−/−^* Mice

**DOI:** 10.3389/fneur.2017.00455

**Published:** 2017-08-30

**Authors:** Sandro M. Krieg, Florian Voigt, Pascal Knuefermann, Carsten Jürgen Kirschning, Nikolaus Plesnila, Florian Ringel

**Affiliations:** ^1^Department of Neurosurgery, Technische Universität München, Munich, Germany; ^2^Institute for Surgical Research, University of Munich Medical Center, Ludwig-Maximilians-Universität München, Munich, Germany; ^3^Department of Anesthesiology and Intensive Care Medicine, University Hospital Bonn, Bonn, Germany; ^4^Institute of Medical Microbiology, University of Duisburg-Essen, Essen, Germany; ^5^Institute for Stroke and Dementia Research, University of Munich Medical Center, Ludwig-Maximilians-Universität München, Munich, Germany; ^6^Department of Neurosurgery, University of Mainz, Mainz, Germany

**Keywords:** toll-like receptors, brain edema, intracranial pressure, secondary brain damage, traumatic brain injury

## Abstract

Danger-associated molecular patterns are released by damaged cells and trigger neuroinflammation through activation of non-specific pattern recognition receptors, e.g., toll-like receptors (TLRs). Since the role of TLR2 and 4 after traumatic brain injury (TBI) is still unclear, we examined the outcome and the expression of pro-inflammatory mediators after experimental TBI in *Tlr2/4^−/−^* and wild-type (WT) mice. *Tlr2/4^−/−^* and WT mice were subjected to controlled cortical injury and contusion volume and brain edema formation were assessed 24 h thereafter. Expression of inflammatory markers in brain tissue was measured by quantitative PCR 15 min, 3 h, 6 h, 12 h, and 24 h after controlled cortical impact (CCI). Contusion volume was significantly attenuated in *Tlr2/4^−/−^* mice (29.7 ± 0.7 mm^3^ as compared to 33.5 ± 0.8 mm^3^ in WT; *p* < 0.05) after CCI while brain edema was not affected. Only interleukin (IL)-1β gene expression was increased after CCI in the *Tlr2/4^−/−^* relative to WT mice. Inducible nitric oxide synthetase, TNF, IL-6, and COX-2 were similar in injured WT and *Tlr2/4^−/−^* mice, while the increase in high-mobility group box 1 was attenuated at 6 h. TLR2 and 4 are consequently shown to potentially promote secondary brain injury after experimental CCI *via* neuroinflammation and may therefore represent a novel therapeutic target for the treatment of TBI.

## Introduction

Head injury is a major public health problem as it is the most frequent cause of death and disability in young adults and therefore produces considerable demands on health services (1). In the pathophysiology of TBI, primary and secondary brain damage needs to be differentiated. The primary tissue damage in the moment of injury occurs due to mechanical forces to the brain parenchyma. Nevertheless, a secondary expansion of the primary lesion during the following hours and days caused by molecular events initiated by the primary lesion leads to propagation of the tissue damage and is an important predictor of clinical outcome (2). As a matter of course, we are unable to reverse and reduce the instant primary brain damage, but delayed secondary loss of tissue is a target for the therapy of TBI and its sequelae for many years. Though several clinical management strategies to reduce secondary brain damage are summarized in guidelines for the treatment of traumatic brain injury (TBI), the efforts to directly target molecular events by neuroprotective drugs failed so far (3–5).

Recently, neuroinflammation has repeatedly been shown to be a major pathophysiological mechanism causing cell death and therefore the aggravation of various cerebral pathologies including TBI (6, 7). Consequently, its reduction might reduce secondary brain damage effectively. Similar to infectious disease-driven disturbance of homeostasis, e.g., by tissue debris, trauma induces activation of the innate immune system albeit in the absence of exogenous compounds. Accordingly, aseptic physical injury to the brain has been shown to elicit an acute immune response that involves activation of resident microglial cells and increased production of pro- and anti-inflammatory cytokines, chemokines, and adhesion molecules (8). Non-infectious inflammatory reactions occur toward released danger-associated molecular patterns, which alert the innate immune system through various pattern recognition receptors (PRRs) (9). Toll-like receptors (TLRs) are a family of PRRs involved in the recognition of invariant molecular motifs of bacteria, fungi, parasites, and viruses and are highly conserved (10). According to recent literature, TLR2 and TLR4, expressed by both microglia and astrocytes, recognize the widest array of binding partners, such as peptidoglycan, lipoproteins, and lipoteichoic acid as well as lipopolysaccharide (LPS) from the outer cell wall of Gram-negative bacteria, respectively (11, 12). Though they also recognize endogenous danger signals such as high-mobility group box 1 (HMGB1) or hyaluronan fragments, or heparan sulfate, or fibrinogen, respectively (13–15).

TLR2 and 4 activation in aseptic brain injury induces a neuroinflammatory cascade by induction of pro-inflammatory cytokines and thereby might induce secondary tissue damage (16). Previous studies showed an upregulation of TLR2 and 4 following experimental ischemic stroke, while genetic deletion of TLR2, 4, and 9 attenuated tissue damage (14). In this study, we therefore hypothesize that synergistic activation of TLR2 and 4 may well contribute to post-TBI when using a clinically more relevant TBI model. Therefore, we used TLR2 and 4 double-knockout mice and investigated secondary necrosis growth, brain edema formation, and expression of inflammatory markers following controlled cortical impact (CCI).

To investigate most pro-inflammatory pathways, interleukin (IL)-1β, HMGB1, inducible nitric oxide synthetase (iNOS), TNF, IL-6, and COX-2 expression were investigated.

## Materials and Methods

### Animals

We used male and female *Tlr2/4^−/−^* mice and their wild-type (WT) littermates, which were backcrossed for nine times to the C57/BL6 background (20–28 g) (17). All mice had access to water and food *ad libitum*. All experiments were performed in agreement with the guidelines of the animal care institutions of the Technical University of Munich and the University of Munich and approved by the Government of Upper Bavaria (protocol number 118/05). The following group sizes were used:

– Brain water: *n* = 7–8 animals per group, two groups
– Contusion volume: *n* = 5 animals per group, three groups
– PCR: *n* = 5 animals per group, seven groups.

### Anesthesia

Anesthesia was induced in an isoflurane chamber (4%) within 2 min and was maintained by 2% isoflurane, 30% O_2_, and 68% N_2_O applied by a facemask. A feedback-controlled heating pad (FHC, Bowdoinham, ME, USA) maintained body temperature at 37°C.

### Controlled Cortical Impact

After the animals were anesthetized and fixed in a stereotactic frame by a nose clamp, a median incision of the scalp and a craniotomy of 4 mm × 4 mm were performed postero-lateral to the bregma without lacerating the dura mater. CCI (Mouse-Katjuscha 2000, L. Kopacz, University of Mainz, Germany) was performed for 150 ms perpendicular to the dura mater with a diameter of 3 mm, a velocity of 8 m/s, and a dislocation of the brain of 1 mm as described earlier; in previous works, physiological measurements did not show any side effects such as decrease oxygen saturation or apnea (18, 19). Moreover, no animal in this series died before finishing the experiments. The craniotomy was closed by adapting the bone flap with tissue glue (Histoacryl^®^, Braun-Melsungen, Melsungen, Germany), and the scalp was sutured with non-resorbable suture. Mice were kept in an incubator heated to 35°C until recovery of spontaneous motor activity. The procedure was performed under sterile conditions to prevent bacterial contamination.

### Determination of Brain Water Content (BWC)

Twenty-four hours after CCI, mice were sacrificed under deep isoflurane anesthesia, by cervical dislocation and brains were removed. Olfactory bulb, cerebellum, and brain stem were removed, and hemispheres were divided with a brain matrix (Kent Scientific, USA). The brains were immediately weighted in wet state (ww), dried at 110°C for 24 h, and dry weight (dw) was measured. BWC was then calculated by the following formula: (ww − dw)/ww × 100.

### Quantification of Contusion Volume

Mice underwent cervical dislocation 15 min or 24 h after trauma under deep isoflurane anesthesia. Brains were removed, instantly frozen in dry ice, and kept at −20°C until further analysis. Coronal frozen sections with a thickness of 10 µm were sliced every 500 µm with a cryostat (CryoStar HM 560, Microm, Germany) and Nissl stained (20). The contused area was then measured on the digital photographs using standard image analysis software (Olympus DP-soft, Germany) by an investigator blinded to the genotype of the animals. Contusion volume (*V*) was calculated as described earlier (21).

### Reverse Transcriptase Real-time Polymerase Chain Reaction (RT-qPCR)

15 min, 3 h, 6 h, 12 h, and 24 h after CCI, mice (five animals per time point) were re-anesthetized with isoflurane, sacrificed, and brains were removed under sterile conditions. This was also done in non-injured mice and in another group 6 h after sham-operation, which had undergone anesthesia, scalp, and skull opening as well as skull and scalp closing without applying CCI.

Total RNA from whole murine brains was then isolated with the guanidinum thiocyanate method (22). Reverse transcription was performed using “High Capacity cDNA Reverse Transcription Kit” (Applied Biosystems, Darmstadt, Germany). 25 µl RNA aliquots were used in 50 µl reaction mixtures containing 5 µl 10× reverse transcriptase buffer, 5 µl 10× random hexamer primers, 2.5 µl “Multi Scribe Reverse Transcriptase” (50 U/μl), 2 µl 25× dNTP mixture, and 10.5 µl nuclease-free water.

For quantitative RT-PCR, 16.625 ng of single-stranded cDNA was mixed with supplied 2× TaqMan^®^ Gene Expression Master Mix (Applied Biosystems, Carlsbad, CA, USA) and 2.22 µl TaqMan^®^ Gene Expression Assay to a final volume of 33.625 µl. The following TaqMan^®^ Gene Expression Assays were used for this investigation (Table 1). For amplification, 10 µl of the reaction mixture was used in a 384-well optical reaction plate. Each sample underwent 40 cycles of amplification (15 s 95°C, 60 s 60°C) on an Applied Biosystems 7900HT Fast Real-Time PCR System using absolute quantification. The results were adjusted to the housekeeping gene GAPDH. Relative quotients (RQ) of gene expression comparing control mice with operated mice at different time points were calculated with Microsoft Excel. RQ results were analyzed using the GraphPad Prism 4.05 (GraphPad Software, San Diego, CA, USA).

**Table 1:** Used primers for the PCR.

Gene	Amplicon size (bp)	Assay ID
Glyeraldehyde-3-phosphate	107	Mm99999915_g1
Tumor necrosis factor	81	Mm00443258_m1
Interleukin-1β	99	Mm99999061_mH
Interleukin-6	67	Mm01210732_g1
Prostaglandin-endoperoxide synthase 2 (COX-2)	80	Mm00478374_m1
Inducible nitric oxide synthetase	70	Mm00440485_m1
High-mobility group box 1	158	Mm00849805_gH

### Experimental Design

At first, we investigated post-traumatic brain edema formation (*n* = 7–8 per group) and secondary contusion expansion (*n* = 5 per group) 24 h after CCI in *Tlr2/4^−/−^* and WT mice. For measuring secondary contusion expansion, another five animals were sacrificed 15 min after CCI to measure primary contusion volume. In these experiments, all WT animals were randomly assigned to each group, and the investigator who performed animal surgery was blinded in terms of the strain’s genetic characteristics.

For investigation of mRNA expression in WT mice, we examined seven groups each consisting five animals: one group of non-injured mice, one group sacrificed 6 h after sham-operation, and five other groups in which brains were removed 15 min, 3 h, 6 h, 12 h, or 24 h after CCI. All animals were randomly assigned to the respective group.

For investigation of mRNA expression in TLR2/4 double-deficient animals, we examined five groups each consisting of five animals: one group of animals 6 h after sham-operation, and four other groups in which brains were removed 3, 6, 12, and 24 h after CCI. Again, all animals were randomly assigned to the respective group.

### Statistical Analysis

Measurements over time (mRNA expression) were tested versus baseline with Friedman Repeated Measures analysis of variance (ANOVA) on Ranks followed by Student–Newman–Keuls All Pairwise Multiple Comparison Procedure as *post hoc* test.

Differences between groups were tested by the Kruskal–Wallis test for non-parametric one-way ANOVA followed by Student–Newman–Keuls All Pairwise Multiple Comparison Procedure or Dunn’s test as *post hoc* test. Differences between two groups were tested using the Mann–Whitney–Wilcoxon test for multiple comparisons on ranks for independent samples (SigmaStat 3.5, Jandel Scientific, Erkrath, Germany). *p* < 0.05 was considered significant. All results are presented as mean ± SEM.

## Results

### Brain Water Content

In WT mice, TBI significantly increased BWC of the traumatized right hemisphere to 80.9 ± 0.5% (*p* < 0.001), while the BWC of the contralateral hemisphere did not change significantly compared to non-traumatized animals (78.5 ± 0.2%) (data not shown). In *Tlr2/4^−/−^* animals, post-traumatic BWC was similar to WT mice in the traumatized (80.3 ± 0.2% in TLR2/4*^−/−^* versus 80.9 ± 0.5% in WT animals; *p* = 0.573) as well as in the non-traumatized contralateral hemisphere (78.3 ± 0.4% in TLR2/4*^−/−^* versus 78.5 ± 0.2% in WT animals; not significant). Thus, post-traumatic BWC was not significantly altered in *Tlr2/4^−/−^* animals.

### Contusion Volume 24 h after CCI

**Figure 1:**
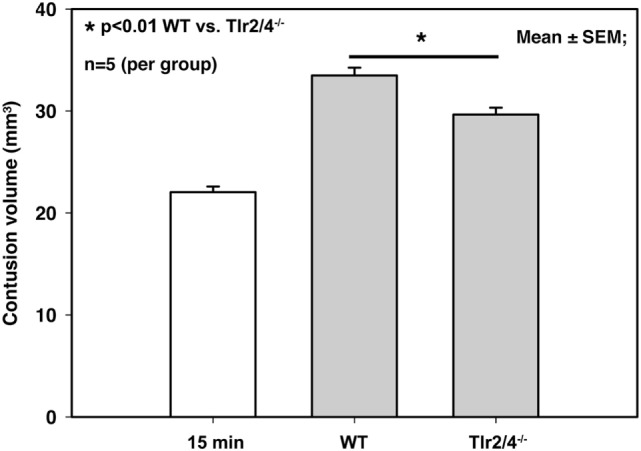
Contusion volume 24 h after controlled cortical impact in wild-type (WT) and Tlr2/4^−/−^ mice. Contusion volume is significantly reduced in Tlr2/4^−/−^ mice (mean ± SEM; *n* = 5 animals per group; *p* < 0.01).

*Tlr2/4^−/−^* animals as well as WT mice showed a significant increase of contusion volume 24 h after trauma compared to the primary brain damage in mice, which were sacrificed 15 min after CCI (22.0 ± 0.6 mm^3^ 15 min after trauma versus 33.5 ± 0.8 mm^3^ in WT mice and 29.7 ± 0.7 mm^3^ in TLR2/4*^−/−^* animals; *p* < 0.001). Contusion volume 24 h after trauma was significantly smaller in *Tlr2/4^−/−^* mice (29.7 ± 0.7 mm^3^) compared to WT animals (33.5 ± 0.8 mm^3^, *p* < 0.01), which represents a reduction of secondary contusion growth by 34% (Figure 1).

### RNA Expression

**Figure 2:**
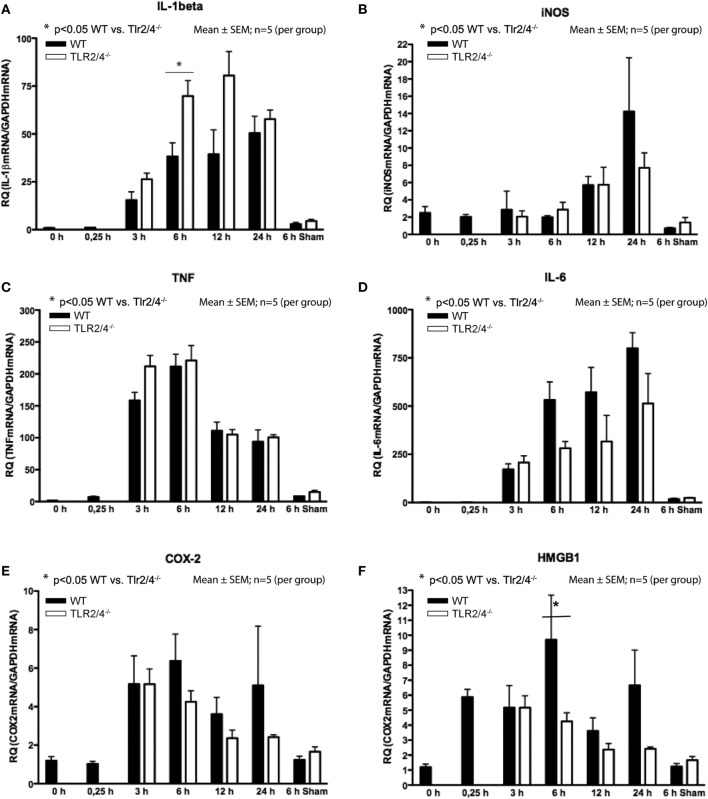
Expression of mRNA after different time points following trauma in wild-type (WT) (black) and Tlr2/4^−/−^ mice (white). Values are expressed relatively to glyceraldehyde-3-phosphate dehydrogenase (GAPDH) as housekeeping gene (mean ± SEM; *n* = 5 animals per group). **(A)** Interleukin (IL)-1beta. **(B)** Inducible nitric oxide synthetase (iNOS). **(C)** TNF. **(D**) IL-6. **(E)** COX-2. **(F)** High-mobility group box (1HMGB1).

Reverse-transcriptase real-time polymerase chain reaction analysis demonstrated a differential expression TLR2 and 4 and IL-1β, iNOS, TNF-α, IL-6, COX-2, and HMGB1. Increased expression of IL-1β was observed from 3 h post-CCI with a maximum increase 12 h (*Tlr2/4^−/−^*) and 24 h (WT) after TBI. Six hours after CCI, we recognized a 1.8-fold higher expression of IL-1β mRNA in *Tlr2/4^−/−^* mice compared to WT animals (*p* < 0.05, Figure 2A).

In respect to the expression of iNOS mRNA, no significant difference between *Tlr2/4^−/−^* and WT mice was observed. But, in *Tlr2/4^−/−^* as well as WT mice a significant (2.3-fold) increase of iNOS expression levels was observed already at 12 h after experimental TBI and was maximal (5.7-fold, WT; 3.1-fold, TLR2/4^−/−^) at the 24 h post-CCI time point (*p* < 0.05, Figure 2B).

Expression of TNF mRNA was increased already 3 h after trauma and maximal (137-fold, WT; 141-fold, TLR2/4^−/−^) 6 h after CCI (Figure 2C). It did not differ in *Tlr2/4^−/−^* and WT mice to a significant degree at any time point. IL-6 mRNA accumulation did also not differ significantly in *Tlr2/4^−/−^* as compared to WT brains at any time point as well.

Expression of IL-6 mRNA was already increased 3 h after CCI with a maximum of 799-fold (WT) and 513-fold (*Tlr2/4^−/−^*) increase 24 h after trauma as compared to controls (Figure 2D).

COX-2 mRNA expression increased 3 h post-CCI and reached maxima of 3.6- and 5.3-fold 3 and 6 h after trauma, respectively, without significant differences between the groups (Figure 2E).

High-mobility group box 1 mRNA expression was increased 4.9-fold at the 15 min time point after experimental TBI already but declined toward 12 h post-CCI (Figure 2F). Moreover, 6 h after trauma, we observed a 2.3-fold higher HMGB1 mRNA expression in WT compared with *Tlr2/4^−/−^* animals (*p* < 0.05).

We observed significantly increased IL-1β, TNF, and IL-6 mRNA expression in sham-operated animals. Still, the mRNA levels remained far below the levels in mice 6 h after CCI. No differences in mRNA expression of iNOS, COX-2, and HMGB1 were observed in sham-operated mice compared to non-operated animals and compared to animals sacrificed 15 min after CCI (Figure 2). Although differences did not reach statistical significance, we observed a robust trend toward a higher expression of IL-1β and a lower expression of iNOS, IL-6, COX-2, and HMGB1 in *Tlr2/4^−/−^* as compared to WT mice, especially 6 h after CCI.

## Discussion

Secondary brain damage is the parenchymal damage that develops on top of the primary tissue damage. This study shows that the expansion of the contused brain volume from 22.0 to 33.5 mm^3^ (*p* < 0.001) was apparent within 24 h upon trauma, i.e., a secondary growth of contusion of 52% as previously shown (18). The central finding of this study is that secondary contusion growth is reduced by 34% in *Tlr2/4^−/−^* mice while post-traumatic brain edema formation was not affected. Although the involved mechanisms are complex and interacting, we interpret our finding that TLR2 and 4 might promote TBI-mediated tissue damage by triggering an enhanced neuroinflammatory response resulting in further cell death.

TLR2 as well as TLR4 are expressed on microglia and astrocytes (11). After acute brain injury, an upregulation and/or release of endogenous TLR ligands including fibrinogen, monosodium urate, HSP70, and heparan sulfate fragments has been observed in both humans and animal models (23, 24). Consequently, we and others speculate that endogenous danger signals released from dying cells or extracellular matrix upon acute brain injury might be recognized by TLR2 and TLR4 to initiate inflammatory responses that aggravate secondary brain damage and increase morbidity after TBI (8).

A reduced inflammatory response after TBI has been observed in *Myd88^−/−^* mice, lacking expression of a key adapter molecule mediating most intracellular signaling of TLRs. This *Myd88^−/−^* phenotype was associated with an attenuation of parenchymal damage after brain cold injury (9). Yet, in such a model, the lack of TLR2 and TLR4 expression by *Tlr2/4^−/−^* mice did not impact on neuroinflammation since their phenotype as compared to that of WT animals was unremarkable. Cold injury models, though, do not mirror clinical reality appropriately; therefore, these results need to be interpreted carefully.

Specific substances released by apoptotic and necrotic cells such as uric acid, HMGB1, and endogenous nucleic acids have been implicated as endogenous immunostimulants (25, 26). Due to parenchymal disruption, the destruction of extracellular matrix causes liberation of heparan sulfate, fibrinogen, or hyaluronan fragments, which have been reported to activate TLR4 (13–15). Accordingly, danger signals such as HMGB1 or hyaluronan fragments activate TLR2 (14, 15). TLR4 recognizes LPS, a component of the outer cell wall of Gram-negative bacteria and endogenous substances such as those listed above (13). Lack of TLR2 and 4 does therefore not allow large parts of pro-inflammatory reaction, which seems to have neuroprotective effects. This is also well in accordance with other data on experimental TBI showing TLR2, TLR4, HSP70, and *Myd88* to accumulate in lesioned regions but also the subcortical white matter (27). Pro-inflammatory mediators such as cytokines drive CNS inflammation by inducing chemokines and adhesion molecules, stimulating immune cells and endogenous glial cells, and recruiting immune cells into the cerebral parenchyma (28).

The observed increase in IL-1β expression is well in accordance with other investigation on spinal cord injury indicating an upregulation of IL-1β in TLR4^−/−^ mice (29). In their discussion, Impellizzeri and colleagues argued that the also increased “NF-KB has an important function in the regulation of many genes responsible for the generation of mediators or proteins in secondary inflammation associated with spinal cord injury such as IL-1β, TNF-α, iNOS” as already shown by Verma in 2004 (29, 30). Moreover, Impellizzeri et al. also showed an increase in neutrophil infiltration, which they explained by “pro-inflammatory cytokines (coordinating) other immune cells, attracting them to the site of damage, amplifying it until the insult is eliminated or dampened by immune-suppressing feedback mechanisms as already demonstrated” by Buchanan et al. in 2010 (29, 31). In the CNS, IL-1β reduces glutamate release, induces expression levels of growth factors and modulates neuronal responses to NMDA and glycine (28). After CNS injury, IL-1β is immediately released from microglia and astrocytes (32).

Nitric oxide (NO) affects neurodegeneration and aggravates secondary brain damage after TBI and focal cerebral ischemia (33). Moreover, NO is produced by glial iNOS of which is known to be induced not only by IL-1β but also by IFN-α and TNF (6). In a model of focal cerebral ischemia, iNOS was reduced in *Tlr4^−/−^* mice 24 h after ischemia (33). Like IL-1β, TNF is known to be increased after parenchymal cerebral injury as well (32). TNF involvement has been implicated in various experimental models of TBI as well as in human brain injury where it was shown to be released (4, 34, 35). In these studies, TNF levels were increased particularly within the brain parenchyma rapidly after injury, and its level was associated with the neurological deficit in rodents subjected to experimental TBI (3, 4). We confirmed this rapid upregulation of TNF expression (Figure 2) suggesting that it is released by resident cells of the brain parenchyma rather than by blood-borne leukocytes, which invade the brain later than 12 h after TBI (36). We consequently conclude that TLR2 and 4 are not involved in TNF induction during TBI, although TNF itself has been shown to be crucially involved in microglia activation following TBI (37).

After brain injury, increased levels of IL-6 and its receptor have been measured in the cerebral parenchyma in a range of animal models, in human cerebrospinal fluid, and in the serum of patients after TBI (34). Under physiological circumstances, expression of IL-6 mRNA in the brain is very low, although, in a large number of human CNS disorders, IL-6 expression is severely increased. The main reason is supposed to be induction by central production of inflammatory cytokines. For instance, IL-6 can be induced by IL-1, transforming growth factor-b, TNF, and prostaglandins (6). Thus, IL-6 mRNA expression does not have to be induced through direct cellular activation of TLRs, but can also be activated indirectly through the mentioned mediators. IL-6 was also shown to be highly upregulated after trauma in mice (9).

Another important but different group of mediators of inflammatory reaction are prostaglandins that are generated by COX from arachidonic acid and prostaglandin synthase enzymes. As COX-2 is only stimulated as a response to inflammatory mediators, it served as a measure for prostaglandin metabolism in this study. Although prostaglandins are typically known to be powerful pro-inflammatory molecules, they also include anti-inflammatory properties under special conditions. Post-traumatic pharmaceutical inhibition of COX-2 in rats showed improved neurological outcome (38).

Principally, high-mobility group proteins are chromosomal proteins that are involved in transcription as well as recombination and replication of DNA. In addition, HMGB1 is known to be liberated by brain damage after which it was proofed to have extracellular activity by acting as a chemokine and therefore attracting inflammatory cells. It serves as a novel pro-inflammatory cytokine-like factor, which connects excitotoxicity-induced acute damage processes and delayed inflammatory action in the post-ischemic brain (25, 26, 39). After acute TBI, HMGB1 was reported to be upregulated as well (9) and was implicated as TLR2 and 4 ligand qualifying it as potential key mediator for the induction of neuroinflammation (14). According to our data, HMGB1 is involved in the pathophysiology of TBI, and its release is regulated by TLR2 or 4 activation. We may speculate that the lack of inflammatory cytokine upregulation in *Tlr2/4^−/−^* mice may be one mechanism responsible for attenuated neuronal damage.

Mechanisms, which control the different cytokines, are often linked; TNF stimulates expression of IL-1 and IL-6, whereas IL-1 can induce both IL-6 and TNF. So, after brain damage, primary upregulation of cytokines causes infiltration of further inflammatory mediators to the contusion site, and further cytokine signaling results. Especially HMGB1 seems to be one of the most crucial factors why TLR2/4 double-deficiency causes less neuronal cell death. Whether HMGB1 acts as an endogenous ligand that activates TLR2 or 4 signaling in damaged brain tissue or HMGB1 is released through TLR2/4 activation needs further investigation. It might use both possibilities as part of an amplification pathway.

Whether TLR2 or 4, or the combination of both is involved in the development of secondary brain damage, cannot be answered by this study. Conversely, the study by Yu and Zha tells us that TLR2 is involved in the aggravation of secondary brain damage including a reduction of TNF-α, IL-1β, and IL-6 in Tlr2^−/−^ compared to WT mice (40). Likewise, other groups also found reduced focal lesions in Tlr4^−/−^ mice not only after experimental stroke but also after experimental TBI (41, 42).

### Limitations

Nonetheless, the investigation of later time point, such as 7 or 14 days after trauma, should also be considered in consecutive studies to analyze not only short- but also long-term effects of these immunological alterations (43, 44). In such long-term series, additional behavioral outcomes should also be included which were also not subject to this study.

Moreover, there are always limitations when intending to transfer rodent data to human patients. One major difference is that TBI causes additional issues in humans, which are not present in rodent, e.g., growth or secondary development of intraperanchymal hematomas. Accordingly, we are only able to investigate some issues of human TBI in such models rather than mimicking the whole pathophysiological process.

As mentioned earlier, both, male and female mice, were enrolled in this study. Since there are severe differences between genders concerning TBI outcome in rodents, the distribution among groups was homogenous.

As another limitation, nonetheless, this study only examined the gene expression. A follow-up study investigating the protein expression should be performed.

## Conclusion

Taken together, this study shows that TLR2 and 4 might drive neuroinflammation and are involved in the development of secondary brain damage after experimental TBI. Unfortunately, the current results do not allow us to differentiate between the effects of TLR2, TLR4, or the combination of both.

## Ethics Statement

All procedures performed in studies involving animals were in accordance with the ethical standards of the institutional, the national research committee, and the legislation of Upper Bavaria. All experiments were performed in agreement with the guidelines of the animal care institutions of the Technical University of Munich and the University of Munich and approved by the Government of Upper Bavaria (protocol number 118/05). The following group sizes were used: (1) brain water: *n* = 7–8 animals per group, two groups; (2) contusion volume: *n* = 5 animals per group, three groups; (3) PCR: *n* = 5 animals per group, seven groups.

## Author Contributions

Conception and design of the work: NP and FR. Data acquisition: SK, FV, PK, and CK. Drafting the manuscript: SK, NP, and FR. Data analysis, interpretation of data for the work, critically revising the manuscript, final approval of the version to be published, and agreement to be accountable for all aspects of the work in ensuring that questions related to the accuracy or integrity of any part of the work are appropriately investigated and resolved: all authors.

## Conflict of Interest Statement

SK and FR are consultants for Brainlab AG (Munich, Germany). SK is a consultant for Nexstim Plc. (Helsinki, Finland). Yet, all authors report no conflict of interest affecting the materials or methods used in this study or the findings specified in this paper. The study was completely financed by institutional grants of the departments of the authors.

## Abbreviations

BWC, brain water content; CCI, controlled cortical impact; DAMPs, danger-associated molecular patterns; dw, dry weight; GAPDH, glyceraldehyde-3-phosphate dehydrogenase; HMGB1, high-mobility group box 1; LPS, lipopolysaccharide; PRR, pattern recognition receptors; TBI, traumatic brain injury; TLR, toll-like receptor; WT, wild-type.
